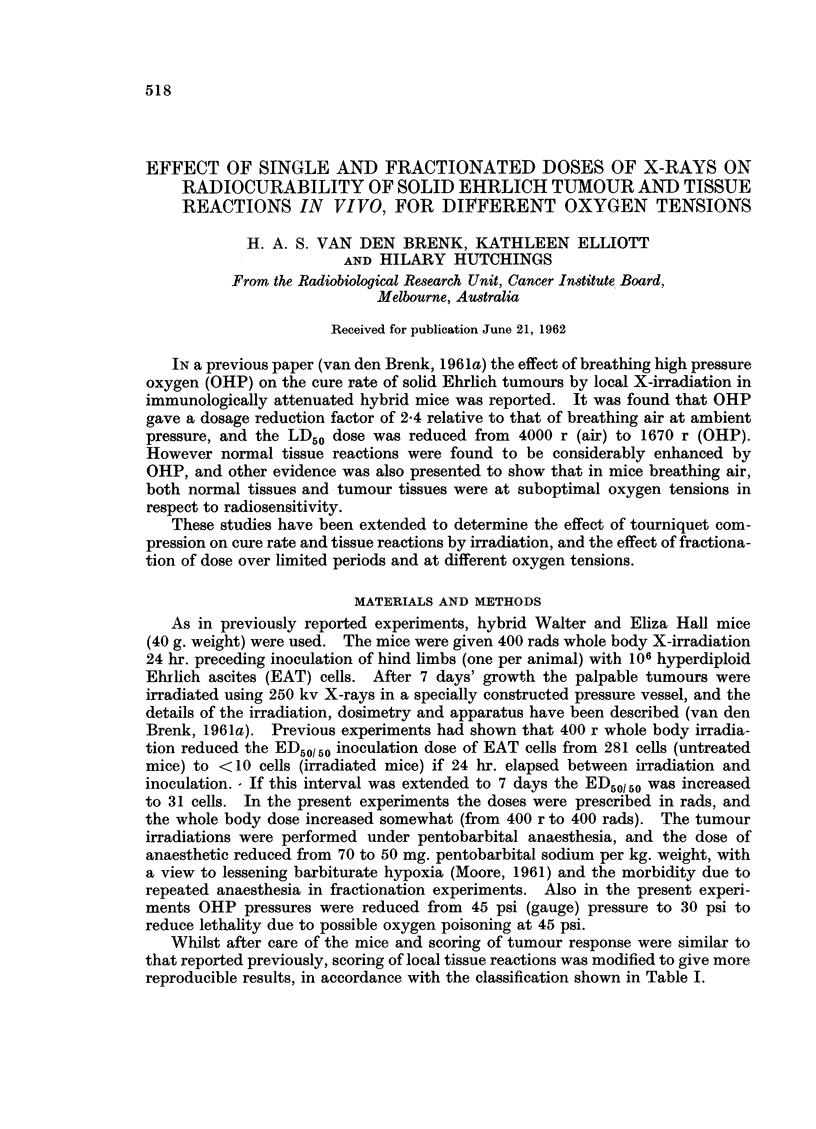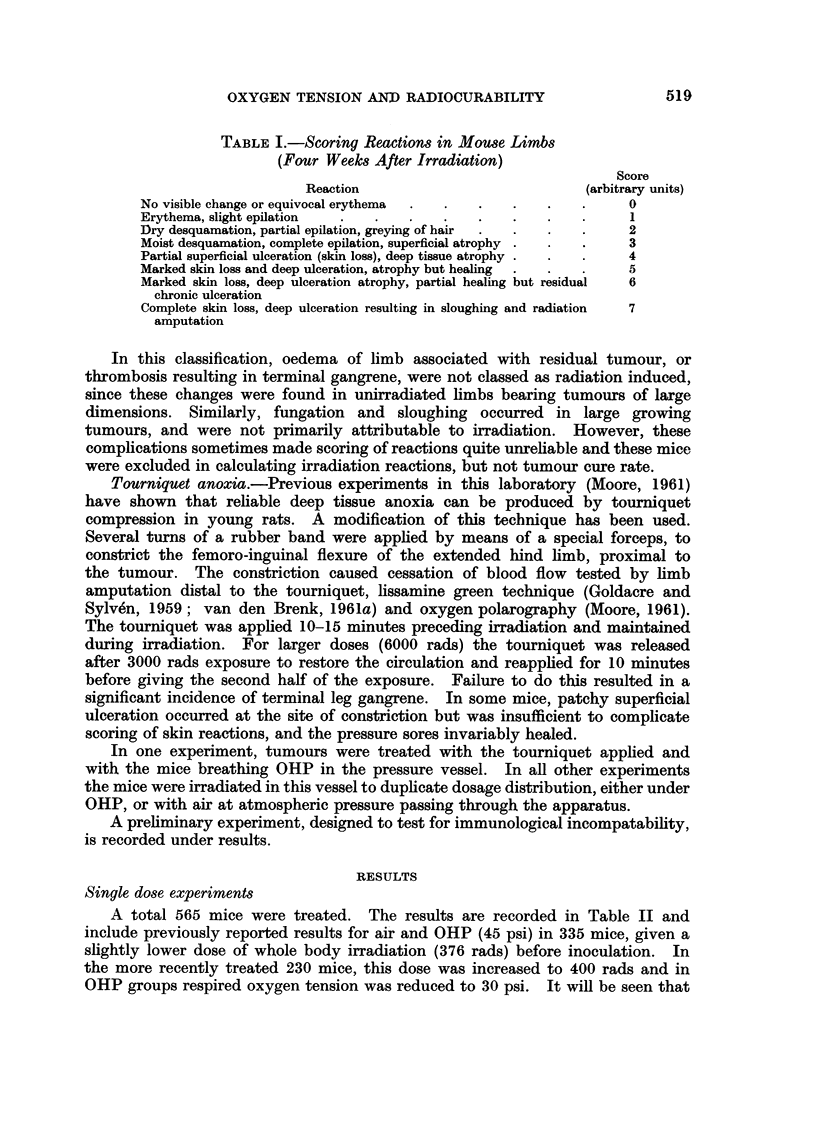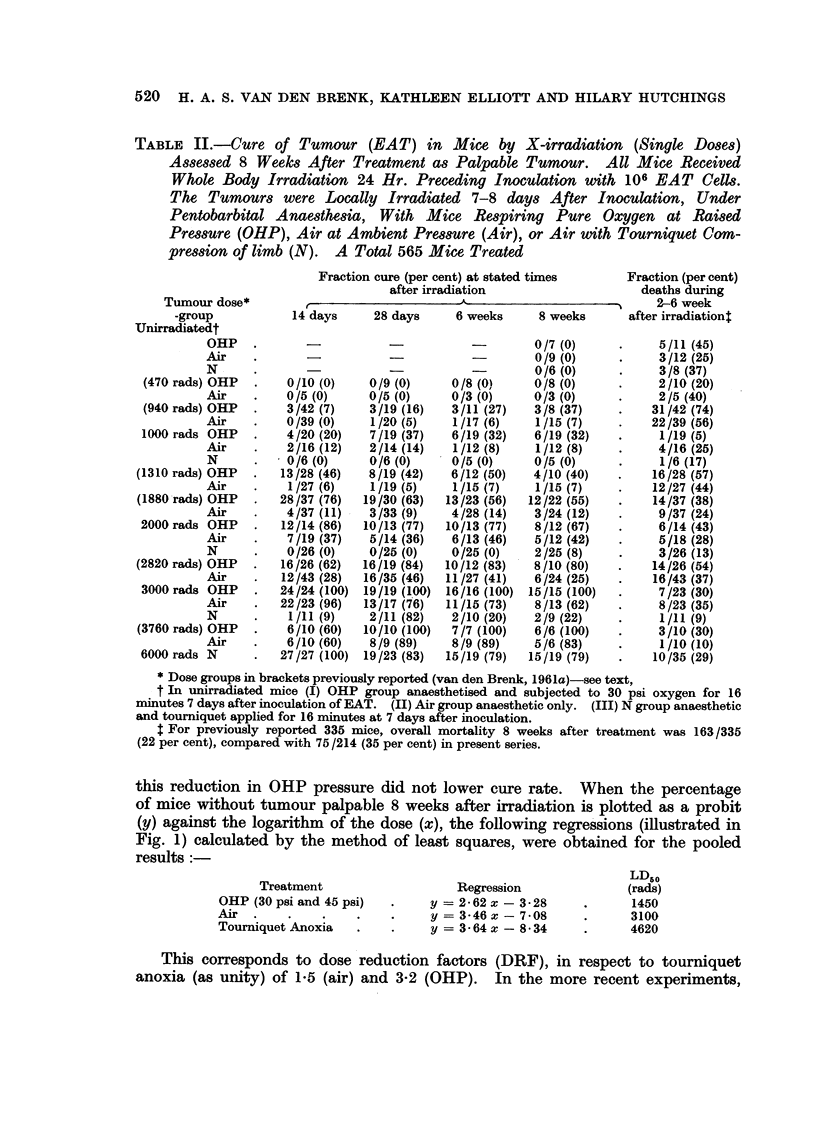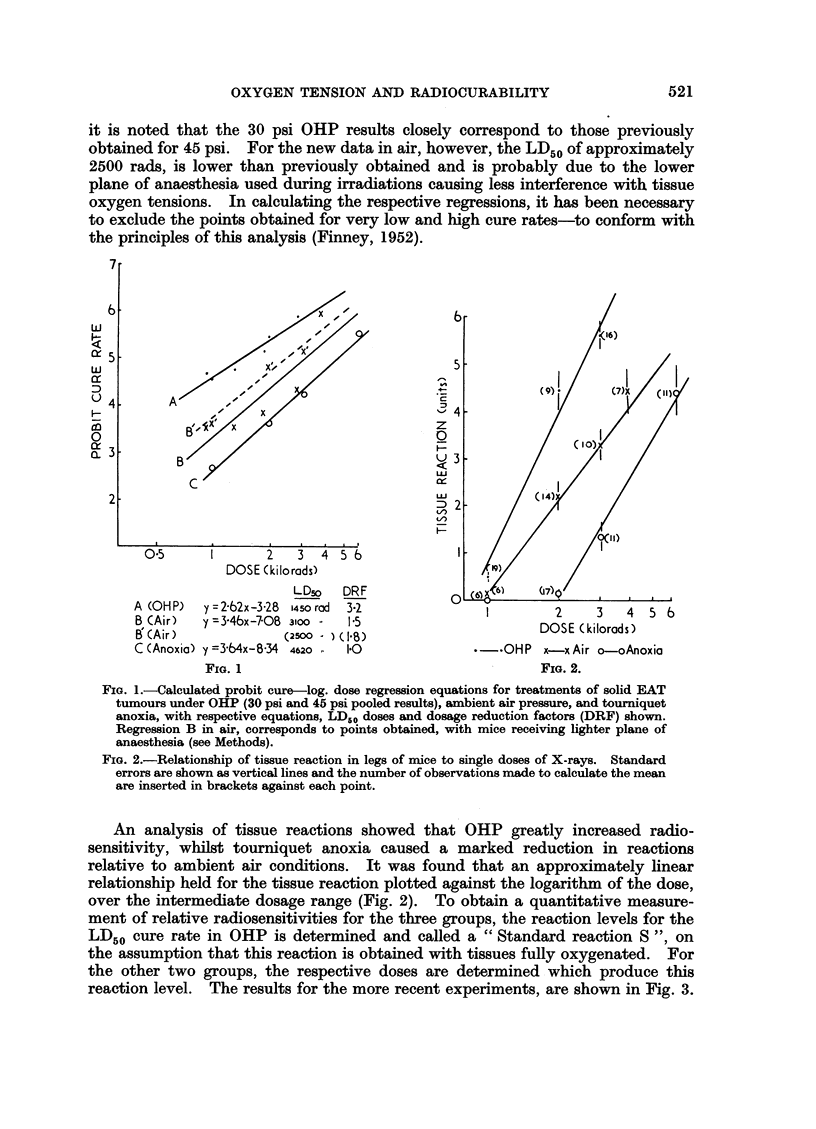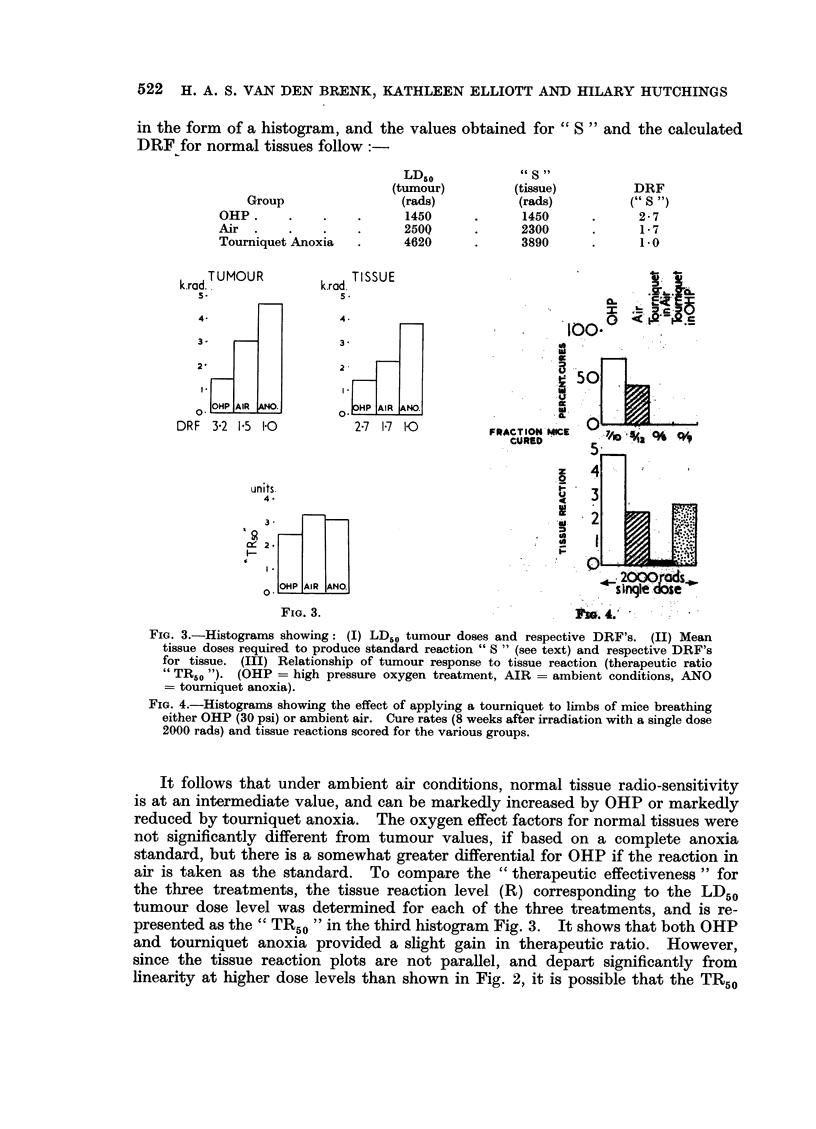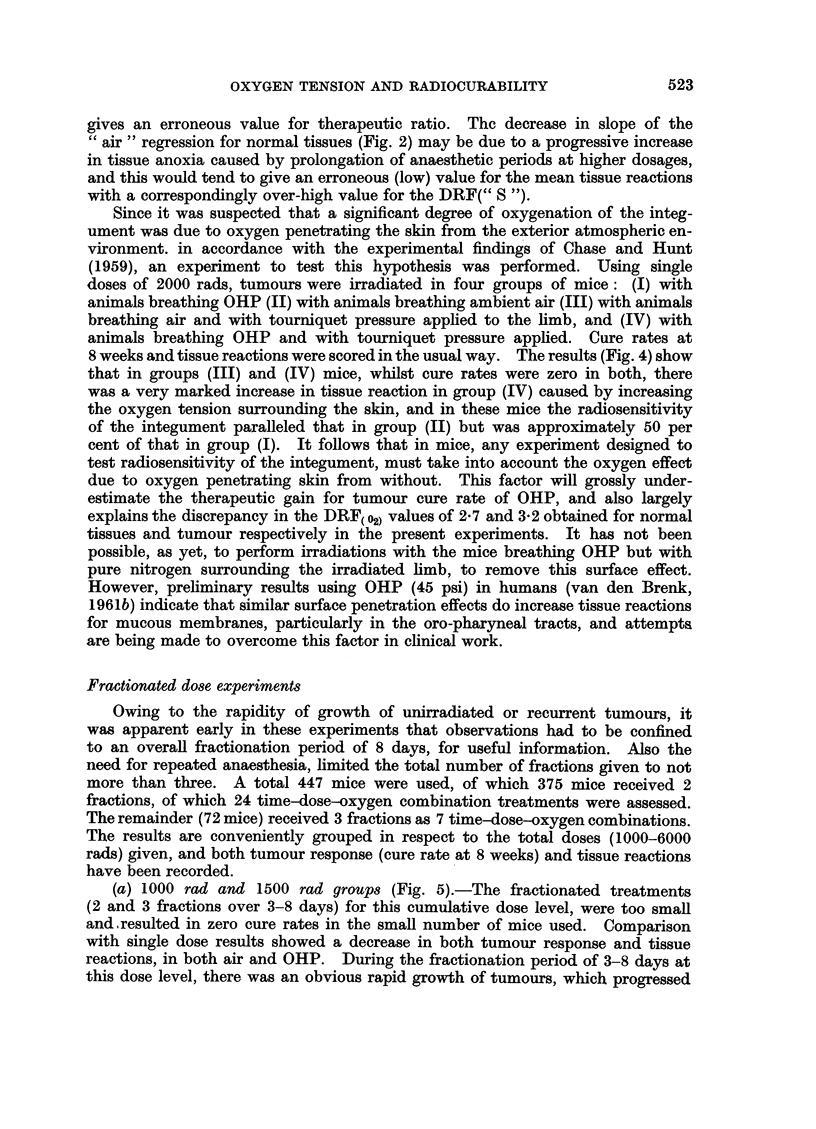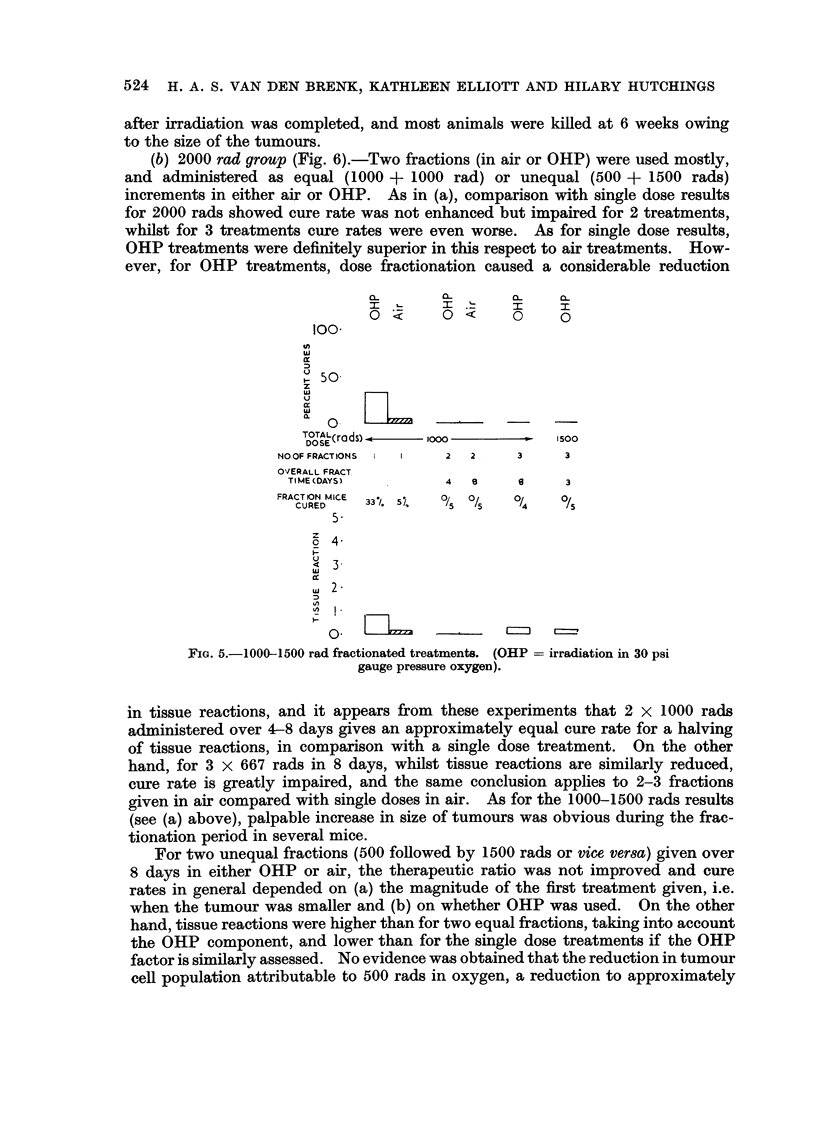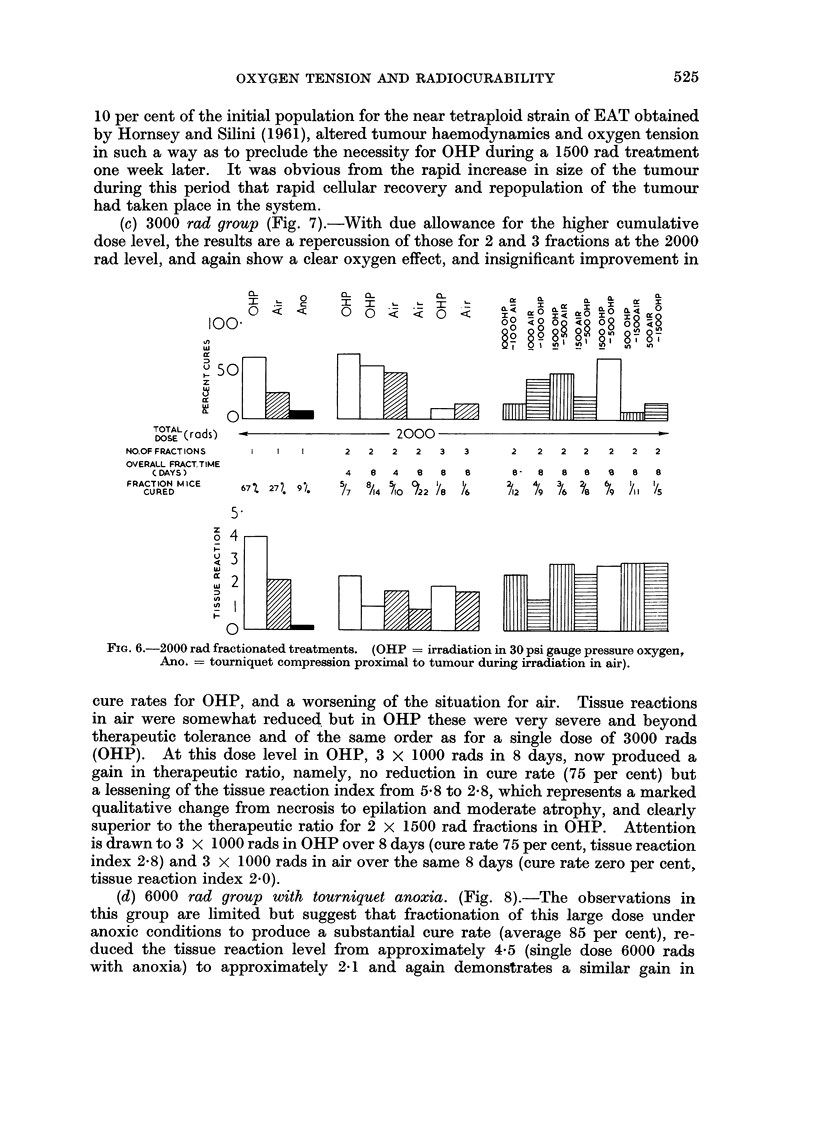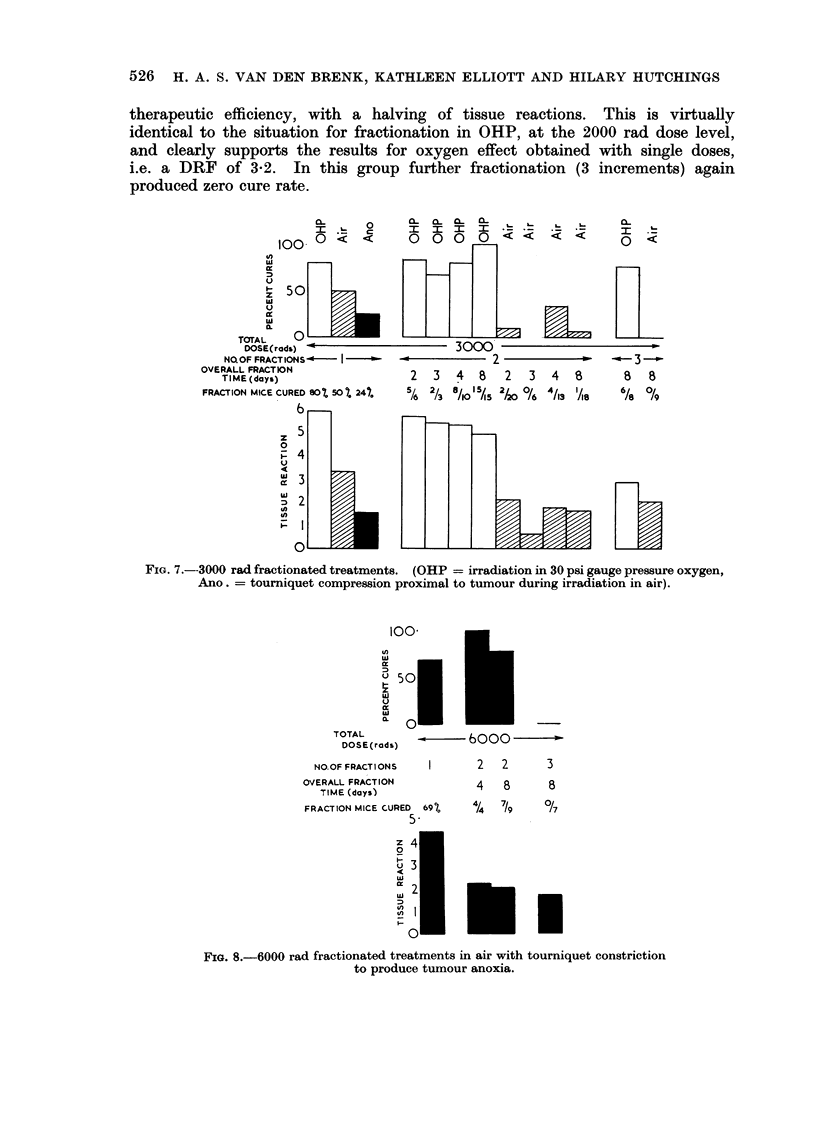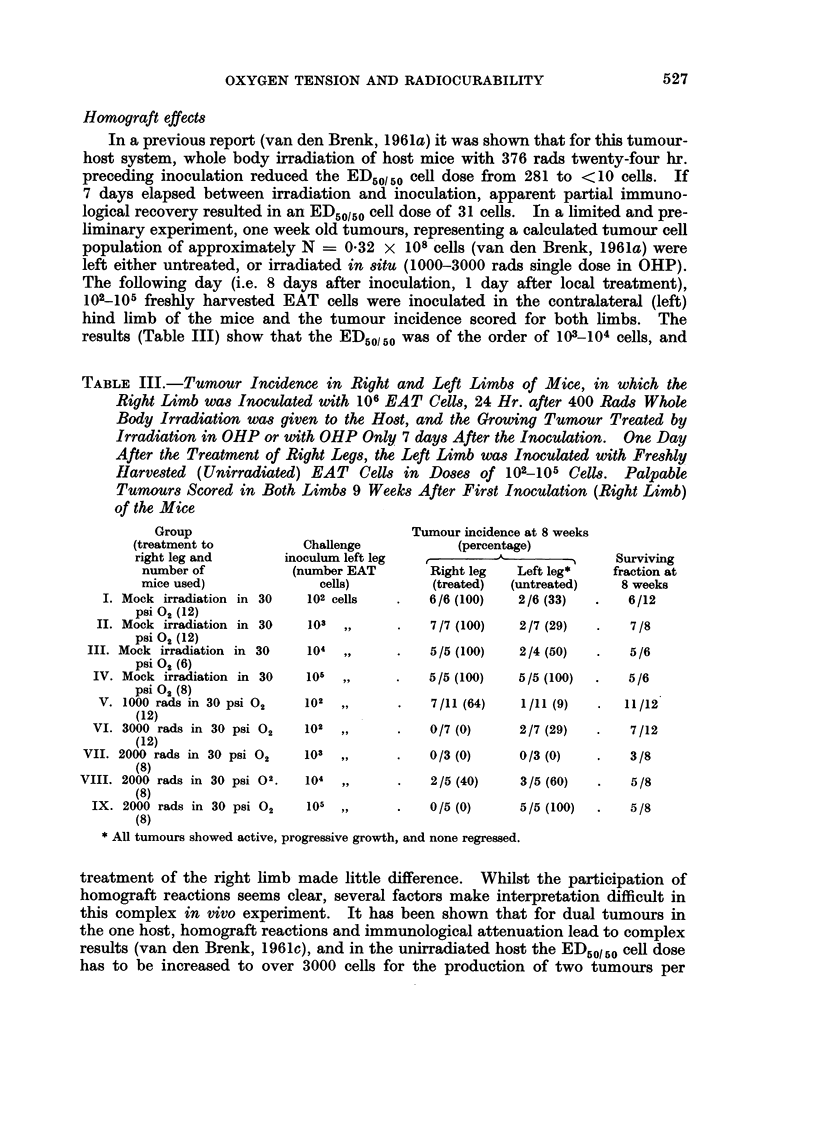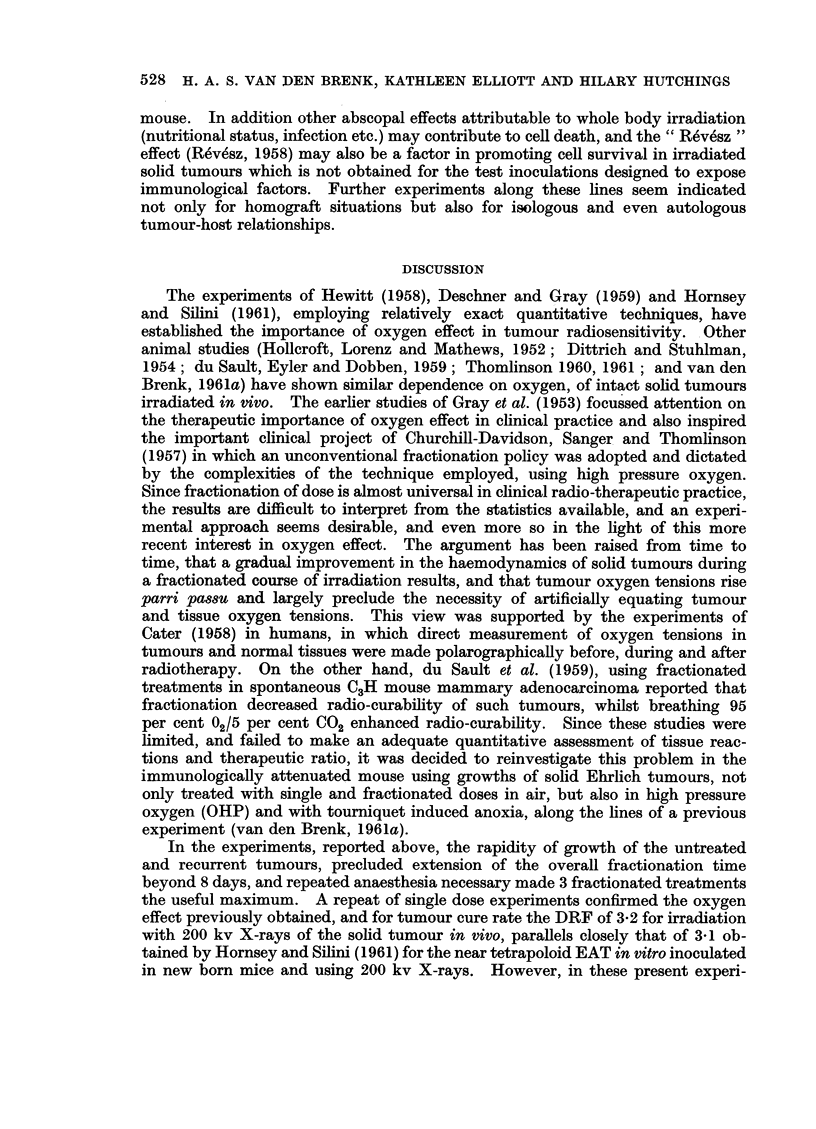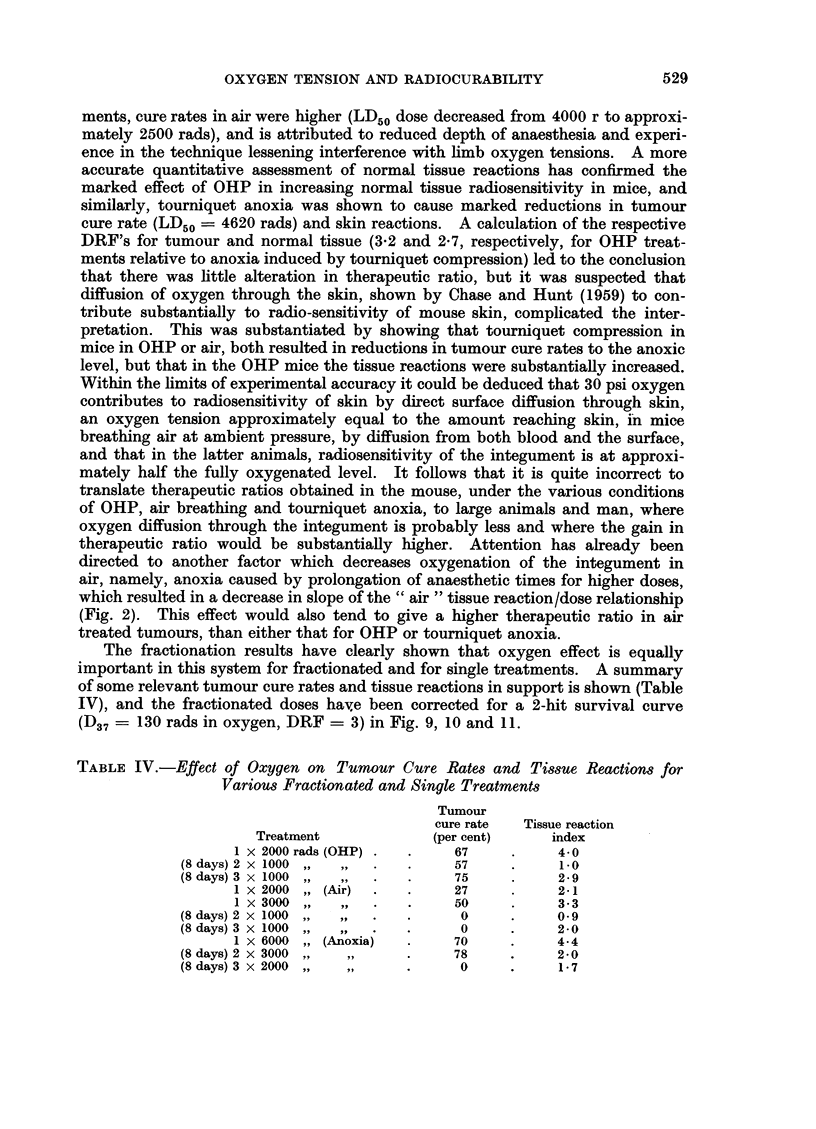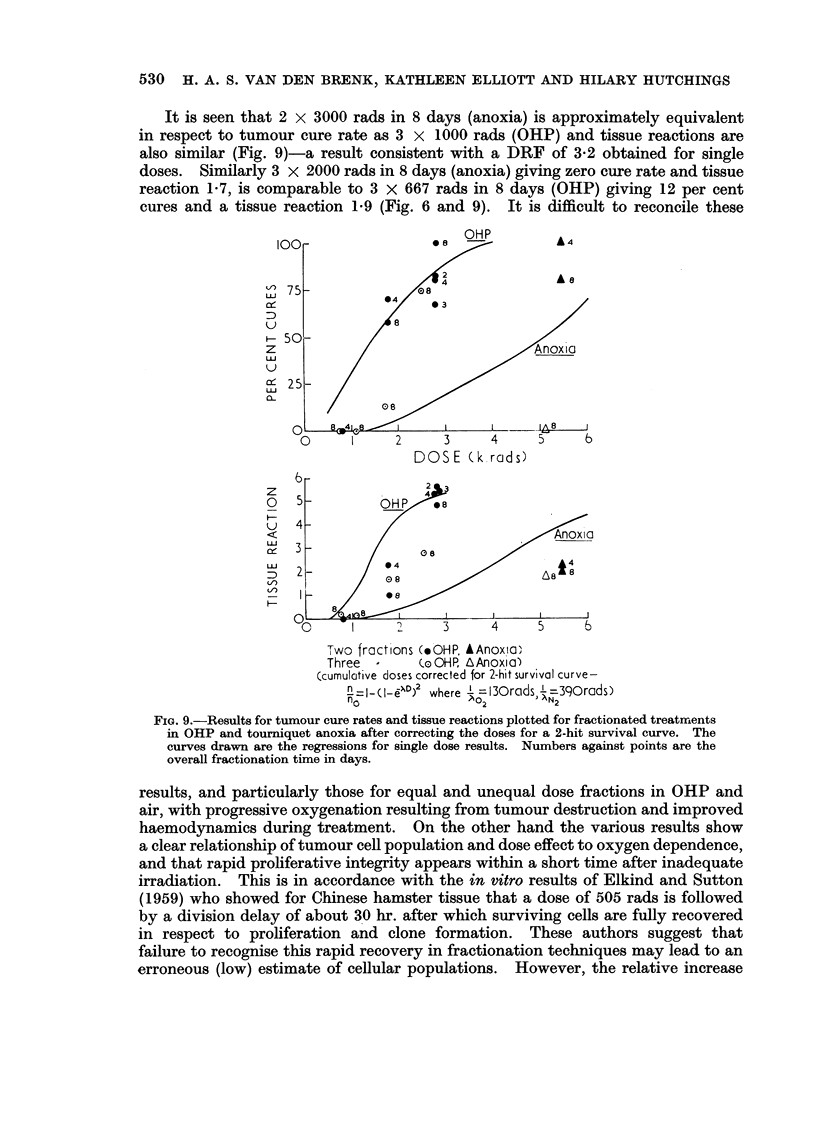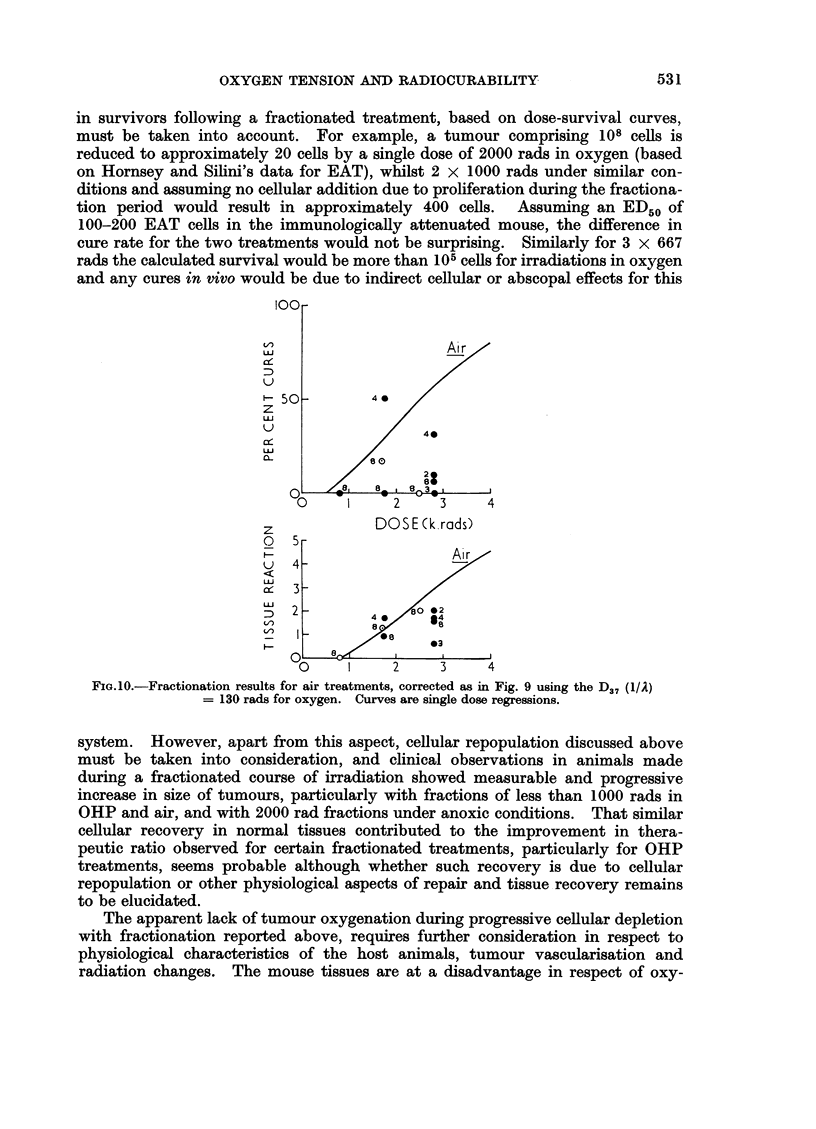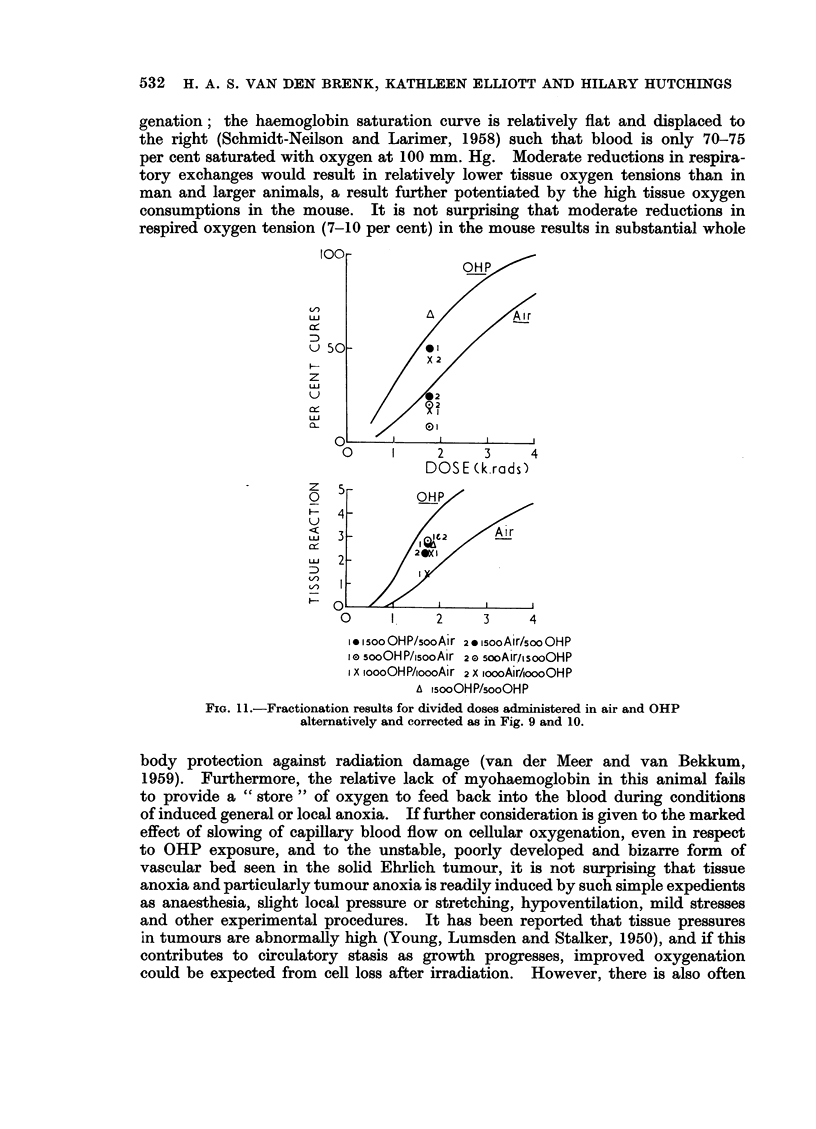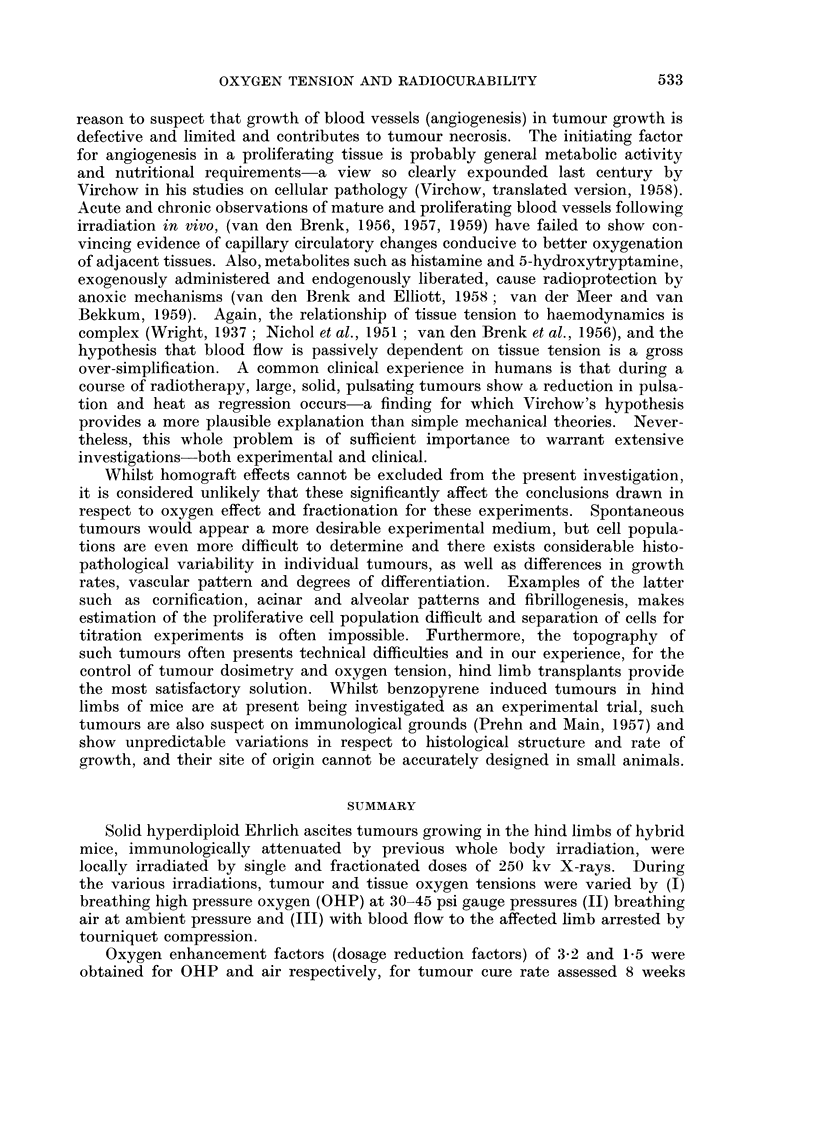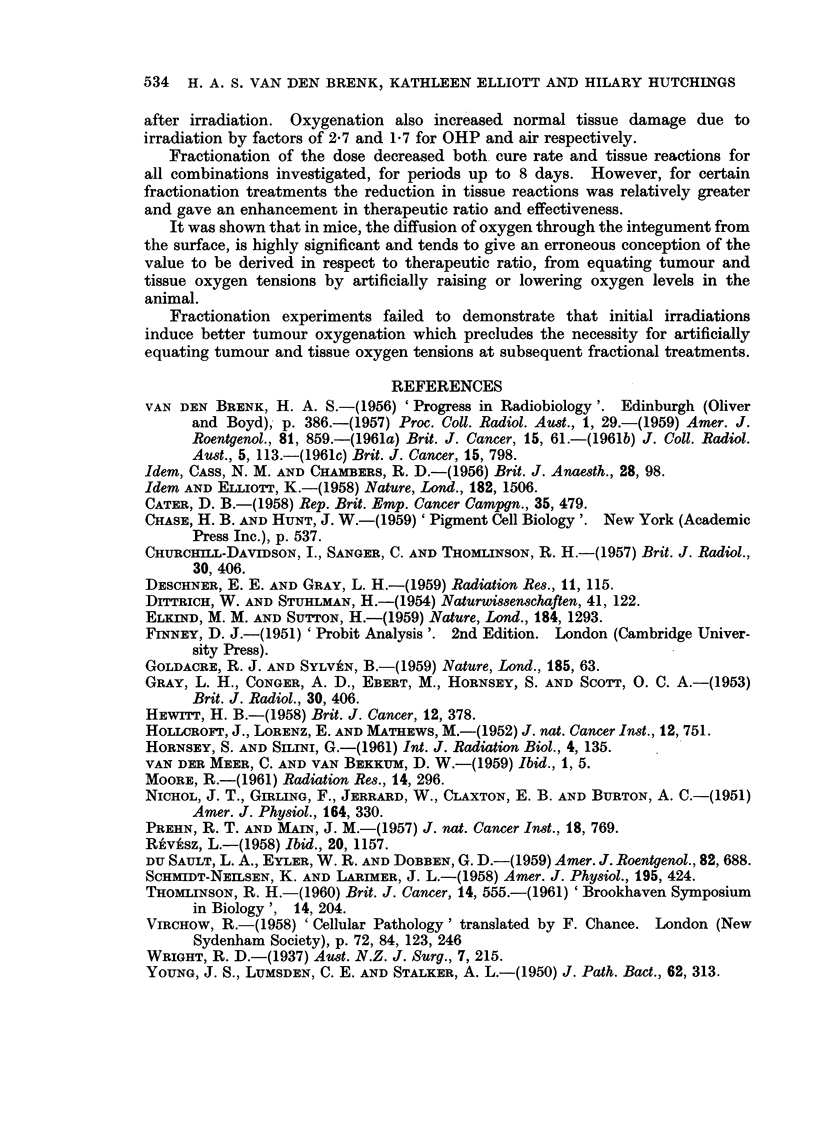# Effect of Single and Fractionated Doses of X-rays on Radiocurability of Solid Ehrlich Tumour and Tissue Reactions In Vivo, for Different Oxygen Tensions

**DOI:** 10.1038/bjc.1962.60

**Published:** 1962-09

**Authors:** H. A. S. van den Brenk, Kathleen Elliott, Hilary Hutchings


### EFFECT OF SINGLE AND FRACTIONATED DOSES OF X-RAYS ON RADIOCURABILITY OF SOLID EHRLICH TUMOUR AND TISSUE REACTIONS *IN VIVO*, FOR DIFFERENT OXYGEN TENSIONS

H. A. S. VAN DEN BRENK, KATHLEEN ELLIOTT AND HILARY HUTCHINGS

*From the Radiobiological Research Unit, Cancer Institute Board, Melbourne, Australia*

Received for publication June 21, 1962

IN a previous paper (van den Brenk, 1961*a*) the effect of breathing high pressure oxygen (OHP) on the cure rate of solid Ehrlich tumours by local X-irradiation in immunologically attenuated hybrid mice was reported. It was found that OHP gave a dosage reduction factor of 2·4 relative to that of breathing air at ambient pressure, and the $LD_{50}$ dose was reduced from 4000 r (air) to 1670 r (OHP). However normal tissue reactions were found to be considerably enhanced by OHP, and other evidence was also presented to show that in mice breathing air, both normal tissues and tumour tissues were at suboptimal oxygen tensions in respect to radiosensitivity.

These studies have been extended to determine the effect of tourniquet compression on cure rate and tissue reactions by irradiation, and the effect of fractionation of dose over limited periods and at different oxygen tensions.

### MATERIALS AND METHODS

As in previously reported experiments, hybrid Walter and Eliza Hall mice (40 g. weight) were used. The mice were given 400 rads whole body X-irradiation 24 hr. preceding inoculation of hind limbs (one per animal) with $10^6$ hyperdiploid Ehrlich ascites (EAT) cells. After 7 days' growth the palpable tumours were irradiated using 250 kv X-rays in a specially constructed pressure vessel, and the details of the irradiation, dosimetry and apparatus have been described (van den Brenk, 1961*a*). Previous experiments had shown that 400 r whole body irradiation reduced the $ED_{50/50}$ inoculation dose of EAT cells from 281 cells (untreated mice) to $<10$ cells (irradiated mice) if 24 hr. elapsed between irradiation and inoculation. If this interval was extended to 7 days the $ED_{50/50}$ was increased to 31 cells. In the present experiments the doses were prescribed in rads, and the whole body dose increased somewhat (from 400 r to 400 rads). The tumour irradiations were performed under pentobarbital anaesthesia, and the dose of anaesthetic reduced from 70 to 50 mg. pentobarbital sodium per kg. weight, with a view to lessening barbiturate hypoxia (Moore, 1961) and the morbidity due to repeated anaesthesia in fractionation experiments. Also in the present experiments OHP pressures were reduced from 45 psi (gauge) pressure to 30 psi to reduce lethality due to possible oxygen poisoning at 45 psi.

Whilst after care of the mice and scoring of tumour response were similar to that reported previously, scoring of local tissue reactions was modified to give more reproducible results, in accordance with the classification shown in Table I.

TABLE I.—*Scoring Reactions in Mouse Limbs (Four Weeks After Irradiation)*

| Reaction | Score (arbitrary units) |
|---|---|
| No visible change or equivocal erythema | 0 |
| Erythema, slight epilation | 1 |
| Dry desquamation, partial epilation, greying of hair | 2 |
| Moist desquamation, complete epilation, superficial atrophy | 3 |
| Partial superficial ulceration (skin loss), deep tissue atrophy | 4 |
| Marked skin loss and deep ulceration, atrophy but healing | 5 |
| Marked skin loss, deep ulceration atrophy, partial healing but residual chronic ulceration | 6 |
| Complete skin loss, deep ulceration resulting in sloughing and radiation amputation | 7 |

In this classification, oedema of limb associated with residual tumour, or thrombosis resulting in terminal gangrene, were not classed as radiation induced, since these changes were found in unirradiated limbs bearing tumours of large dimensions. Similarly, fungation and sloughing occurred in large growing tumours, and were not primarily attributable to irradiation. However, these complications sometimes made scoring of reactions quite unreliable and these mice were excluded in calculating irradiation reactions, but not tumour cure rate.

*Tourniquet anoxia.*—Previous experiments in this laboratory (Moore, 1961) have shown that reliable deep tissue anoxia can be produced by tourniquet compression in young rats. A modification of this technique has been used. Several turns of a rubber band were applied by means of a special forceps, to constrict the femoro-inguinal flexure of the extended hind limb, proximal to the tumour. The constriction caused cessation of blood flow tested by limb amputation distal to the tourniquet, lissamine green technique (Goldacre and Sylvén, 1959; van den Brenk, 1961*a*) and oxygen polarography (Moore, 1961). The tourniquet was applied 10–15 minutes preceding irradiation and maintained during irradiation. For larger doses (6000 rads) the tourniquet was released after 3000 rads exposure to restore the circulation and reapplied for 10 minutes before giving the second half of the exposure. Failure to do this resulted in a significant incidence of terminal leg gangrene. In some mice, patchy superficial ulceration occurred at the site of constriction but was insufficient to complicate scoring of skin reactions, and the pressure sores invariably healed.

In one experiment, tumours were treated with the tourniquet applied and with the mice breathing OHP in the pressure vessel. In all other experiments the mice were irradiated in this vessel to duplicate dosage distribution, either under OHP, or with air at atmospheric pressure passing through the apparatus.

A preliminary experiment, designed to test for immunological incompatability, is recorded under results.

### RESULTS

*Single dose experiments*

A total 565 mice were treated. The results are recorded in Table II and include previously reported results for air and OHP (45 psi) in 335 mice, given a slightly lower dose of whole body irradiation (376 rads) before inoculation. In the more recently treated 230 mice, this dose was increased to 400 rads and in OHP groups respired oxygen tension was reduced to 30 psi. It will be seen that

TABLE II.—*Cure of Tumour (EAT) in Mice by X-irradiation (Single Doses) Assessed 8 Weeks After Treatment as Palpable Tumour. All Mice Received Whole Body Irradiation 24 Hr. Preceding Inoculation with* $10^6$ *EAT Cells. The Tumours were Locally Irradiated 7–8 days After Inoculation, Under Pentobarbital Anaesthesia, With Mice Respiring Pure Oxygen at Raised Pressure (OHP), Air at Ambient Pressure (Air), or Air with Tourniquet Compression of limb (N). A Total 565 Mice Treated*

| Tumour dose* -group | Fraction cure (per cent) at stated times after irradiation: 14 days | 28 days | 6 weeks | 8 weeks | Fraction (per cent) deaths during 2–6 week after irradiation‡ |
|---|---|---|---|---|---|
| Unirradiated† | | | | | |
| OHP | — | — | — | 0/7 (0) | 5/11 (45) |
| Air | — | — | — | 0/9 (0) | 3/12 (25) |
| N | — | — | — | 0/6 (0) | 3/8 (37) |
| (470 rads) OHP | 0/10 (0) | 0/9 (0) | 0/8 (0) | 0/8 (0) | 2/10 (20) |
| Air | 0/5 (0) | 0/5 (0) | 0/3 (0) | 0/3 (0) | 2/5 (40) |
| (940 rads) OHP | 3/42 (7) | 3/19 (16) | 3/11 (27) | 3/8 (37) | 31/42 (74) |
| Air | 0/39 (0) | 1/20 (5) | 1/17 (6) | 1/15 (7) | 22/39 (56) |
| 1000 rads OHP | 4/20 (20) | 7/19 (37) | 6/19 (32) | 6/19 (32) | 1/19 (5) |
| Air | 2/16 (12) | 2/14 (14) | 1/12 (8) | 1/12 (8) | 4/16 (25) |
| N | 0/6 (0) | 0/6 (0) | 0/5 (0) | 0/5 (0) | 1/6 (17) |
| (1310 rads) OHP | 13/28 (46) | 8/19 (42) | 6/12 (50) | 4/10 (40) | 16/28 (57) |
| Air | 1/27 (6) | 1/19 (5) | 1/15 (7) | 1/15 (7) | 12/27 (44) |
| (1880 rads) OHP | 28/37 (76) | 19/30 (63) | 13/23 (56) | 12/22 (55) | 14/37 (38) |
| Air | 4/37 (11) | 3/33 (9) | 4/28 (14) | 3/24 (12) | 9/37 (24) |
| 2000 rads OHP | 12/14 (86) | 10/13 (77) | 10/13 (77) | 8/12 (67) | 6/14 (43) |
| Air | 7/19 (37) | 5/14 (36) | 6/13 (46) | 5/12 (42) | 5/18 (28) |
| N | 0/26 (0) | 0/25 (0) | 0/25 (0) | 2/25 (8) | 3/26 (13) |
| (2820 rads) OHP | 16/26 (62) | 16/19 (84) | 10/12 (83) | 8/10 (80) | 14/26 (54) |
| Air | 12/43 (28) | 16/35 (46) | 11/27 (41) | 6/24 (25) | 16/43 (37) |
| 3000 rads OHP | 24/24 (100) | 19/19 (100) | 16/16 (100) | 15/15 (100) | 7/23 (30) |
| Air | 22/23 (96) | 13/17 (76) | 11/15 (73) | 8/13 (62) | 8/23 (35) |
| N | 1/11 (9) | 2/11 (82) | 2/10 (20) | 2/9 (22) | 1/11 (9) |
| (3760 rads) OHP | 6/10 (60) | 10/10 (100) | 7/7 (100) | 6/6 (100) | 3/10 (30) |
| Air | 6/10 (60) | 8/9 (89) | 8/9 (89) | 5/6 (83) | 1/10 (10) |
| 6000 rads N | 27/27 (100) | 19/23 (83) | 15/19 (79) | 15/19 (79) | 10/35 (29) |

* Dose groups in brackets previously reported (van den Brenk, 1961*a*)—see text,

† In unirradiated mice (I) OHP group anaesthetised and subjected to 30 psi oxygen for 16 minutes 7 days after inoculation of EAT. (II) Air group anaesthetic only. (III) N group anaesthetic and tourniquet applied for 16 minutes at 7 days after inoculation.

‡ For previously reported 335 mice, overall mortality 8 weeks after treatment was 163/335 (22 per cent), compared with 75/214 (35 per cent) in present series.

this reduction in OHP pressure did not lower cure rate. When the percentage of mice without tumour palpable 8 weeks after irradiation is plotted as a probit ($y$) against the logarithm of the dose ($x$), the following regressions (illustrated in Fig. 1) calculated by the method of least squares, were obtained for the pooled results :—

| Treatment | Regression | $LD_{50}$ (rads) |
|---|---|---|
| OHP (30 psi and 45 psi) | $y = 2 \cdot 62\, x - 3 \cdot 28$ | 1450 |
| Air | $y = 3 \cdot 46\, x - 7 \cdot 08$ | 3100 |
| Tourniquet Anoxia | $y = 3 \cdot 64\, x - 8 \cdot 34$ | 4620 |

This corresponds to dose reduction factors (DRF), in respect to tourniquet anoxia (as unity) of 1·5 (air) and 3·2 (OHP). In the more recent experiments, it is noted that the 30 psi OHP results closely correspond to those previously obtained for 45 psi. For the new data in air, however, the $LD_{50}$ of approximately 2500 rads, is lower than previously obtained and is probably due to the lower plane of anaesthesia used during irradiations causing less interference with tissue oxygen tensions. In calculating the respective regressions, it has been necessary to exclude the points obtained for very low and high cure rates—to conform with the principles of this analysis (Finney, 1952).

| | | $LD_{50}$ | DRF |
|---|---|---|---|
| A (OHP) | $y = 2{\cdot}62x - 3{\cdot}28$ | 1450 rad | 3·2 |
| B (Air) | $y = 3{\cdot}46x - 7{\cdot}08$ | 3100 " | 1·5 |
| B' (Air) | | (2500 " ) | (1·8) |
| C (Anoxia) | $y = 3{\cdot}64x - 8{\cdot}34$ | 4620 " | 1·0 |

FIG. 1

FIG. 2.

FIG. 1.—Calculated probit cure—log. dose regression equations for treatments of solid EAT tumours under OHP (30 psi and 45 psi pooled results), ambient air pressure, and tourniquet anoxia, with respective equations, $LD_{50}$ doses and dosage reduction factors (DRF) shown. Regression B in air, corresponds to points obtained, with mice receiving lighter plane of anaesthesia (see Methods).

FIG. 2.—Relationship of tissue reaction in legs of mice to single doses of X-rays. Standard errors are shown as vertical lines and the number of observations made to calculate the mean are inserted in brackets against each point.

An analysis of tissue reactions showed that OHP greatly increased radiosensitivity, whilst tourniquet anoxia caused a marked reduction in reactions relative to ambient air conditions. It was found that an approximately linear relationship held for the tissue reaction plotted against the logarithm of the dose, over the intermediate dosage range (Fig. 2). To obtain a quantitative measurement of relative radiosensitivities for the three groups, the reaction levels for the $LD_{50}$ cure rate in OHP is determined and called a " Standard reaction S ", on the assumption that this reaction is obtained with tissues fully oxygenated. For the other two groups, the respective doses are determined which produce this reaction level. The results for the more recent experiments, are shown in Fig. 3.

in the form of a histogram, and the values obtained for " S " and the calculated DRF for normal tissues follow :—

| Group | $LD_{50}$ (tumour) (rads) | " S " (tissue) (rads) | DRF (" S ") |
|---|---|---|---|
| OHP . . . . | 1450 | 1450 | 2·7 |
| Air . . . . | 2500 | 2300 | 1·7 |
| Tourniquet Anoxia . | 4620 | 3890 | 1·0 |

FIG. 3.

FIG. 4.

FIG. 3.—Histograms showing : (I) $LD_{50}$ tumour doses and respective DRF's. (II) Mean tissue doses required to produce standard reaction " S " (see text) and respective DRF's for tissue. (III) Relationship of tumour response to tissue reaction (therapeutic ratio " $TR_{50}$ "). (OHP = high pressure oxygen treatment, AIR = ambient conditions, ANO = tourniquet anoxia).

FIG. 4.—Histograms showing the effect of applying a tourniquet to limbs of mice breathing either OHP (30 psi) or ambient air. Cure rates (8 weeks after irradiation with a single dose 2000 rads) and tissue reactions scored for the various groups.

It follows that under ambient air conditions, normal tissue radio-sensitivity is at an intermediate value, and can be markedly increased by OHP or markedly reduced by tourniquet anoxia. The oxygen effect factors for normal tissues were not significantly different from tumour values, if based on a complete anoxia standard, but there is a somewhat greater differential for OHP if the reaction in air is taken as the standard. To compare the " therapeutic effectiveness " for the three treatments, the tissue reaction level (R) corresponding to the $LD_{50}$ tumour dose level was determined for each of the three treatments, and is represented as the " $TR_{50}$ " in the third histogram Fig. 3. It shows that both OHP and tourniquet anoxia provided a slight gain in therapeutic ratio. However, since the tissue reaction plots are not parallel, and depart significantly from linearity at higher dose levels than shown in Fig. 2, it is possible that the $TR_{50}$

gives an erroneous value for therapeutic ratio. Thc decrease in slope of the " air " regression for normal tissues (Fig. 2) may be due to a progressive increase in tissue anoxia caused by prolongation of anaesthetic periods at higher dosages, and this would tend to give an erroneous (low) value for the mean tissue reactions with a correspondingly over-high value for the DRF(" S ").

Since it was suspected that a significant degree of oxygenation of the integument was due to oxygen penetrating the skin from the exterior atmospheric environment. in accordance with the experimental findings of Chase and Hunt (1959), an experiment to test this hypothesis was performed. Using single doses of 2000 rads, tumours were irradiated in four groups of mice : (I) with animals breathing OHP (II) with animals breathing ambient air (III) with animals breathing air and with tourniquet pressure applied to the limb, and (IV) with animals breathing OHP and with tourniquet pressure applied. Cure rates at 8 weeks and tissue reactions were scored in the usual way. The results (Fig. 4) show that in groups (III) and (IV) mice, whilst cure rates were zero in both, there was a very marked increase in tissue reaction in group (IV) caused by increasing the oxygen tension surrounding the skin, and in these mice the radiosensitivity of the integument paralleled that in group (II) but was approximately 50 per cent of that in group (I). It follows that in mice, any experiment designed to test radiosensitivity of the integument, must take into account the oxygen effect due to oxygen penetrating skin from without. This factor will grossly underestimate the therapeutic gain for tumour cure rate of OHP, and also largely explains the discrepancy in the $DRF_{(O_2)}$ values of 2·7 and 3·2 obtained for normal tissues and tumour respectively in the present experiments. It has not been possible, as yet, to perform irradiations with the mice breathing OHP but with pure nitrogen surrounding the irradiated limb, to remove this surface effect. However, preliminary results using OHP (45 psi) in humans (van den Brenk, 1961*b*) indicate that similar surface penetration effects do increase tissue reactions for mucous membranes, particularly in the oro-pharyneal tracts, and attempts are being made to overcome this factor in clinical work.

### *Fractionated dose experiments*

Owing to the rapidity of growth of unirradiated or recurrent tumours, it was apparent early in these experiments that observations had to be confined to an overall fractionation period of 8 days, for useful information. Also the need for repeated anaesthesia, limited the total number of fractions given to not more than three. A total 447 mice were used, of which 375 mice received 2 fractions, of which 24 time–dose–oxygen combination treatments were assessed. The remainder (72 mice) received 3 fractions as 7 time–dose–oxygen combinations. The results are conveniently grouped in respect to the total doses (1000–6000 rads) given, and both tumour response (cure rate at 8 weeks) and tissue reactions have been recorded.

(*a*) 1000 *rad and* 1500 *rad groups* (Fig. 5).—The fractionated treatments (2 and 3 fractions over 3–8 days) for this cumulative dose level, were too small and resulted in zero cure rates in the small number of mice used. Comparison with single dose results showed a decrease in both tumour response and tissue reactions, in both air and OHP. During the fractionation period of 3–8 days at this dose level, there was an obvious rapid growth of tumours, which progressed after irradiation was completed, and most animals were killed at 6 weeks owing to the size of the tumours.

(*b*) 2000 *rad group* (Fig. 6).—Two fractions (in air or OHP) were used mostly, and administered as equal (1000 + 1000 rad) or unequal (500 + 1500 rads) increments in either air or OHP. As in (a), comparison with single dose results for 2000 rads showed cure rate was not enhanced but impaired for 2 treatments, whilst for 3 treatments cure rates were even worse. As for single dose results, OHP treatments were definitely superior in this respect to air treatments. However, for OHP treatments, dose fractionation caused a considerable reduction

FIG. 5.—1000–1500 rad fractionated treatments. (OHP = irradiation in 30 psi gauge pressure oxygen).

in tissue reactions, and it appears from these experiments that 2 × 1000 rads administered over 4–8 days gives an approximately equal cure rate for a halving of tissue reactions, in comparison with a single dose treatment. On the other hand, for 3 × 667 rads in 8 days, whilst tissue reactions are similarly reduced, cure rate is greatly impaired, and the same conclusion applies to 2–3 fractions given in air compared with single doses in air. As for the 1000–1500 rads results (see (a) above), palpable increase in size of tumours was obvious during the fractionation period in several mice.

For two unequal fractions (500 followed by 1500 rads or *vice versa*) given over 8 days in either OHP or air, the therapeutic ratio was not improved and cure rates in general depended on (a) the magnitude of the first treatment given, i.e. when the tumour was smaller and (b) on whether OHP was used. On the other hand, tissue reactions were higher than for two equal fractions, taking into account the OHP component, and lower than for the single dose treatments if the OHP factor is similarly assessed. No evidence was obtained that the reduction in tumour cell population attributable to 500 rads in oxygen, a reduction to approximately

10 per cent of the initial population for the near tetraploid strain of EAT obtained by Hornsey and Silini (1961), altered tumour haemodynamics and oxygen tension in such a way as to preclude the necessity for OHP during a 1500 rad treatment one week later. It was obvious from the rapid increase in size of the tumour during this period that rapid cellular recovery and repopulation of the tumour had taken place in the system.

(*c*) 3000 *rad group* (Fig. 7).—With due allowance for the higher cumulative dose level, the results are a repercussion of those for 2 and 3 fractions at the 2000 rad level, and again show a clear oxygen effect, and insignificant improvement in

FIG. 6.—2000 rad fractionated treatments. (OHP = irradiation in 30 psi gauge pressure oxygen, Ano. = tourniquet compression proximal to tumour during irradiation in air).

cure rates for OHP, and a worsening of the situation for air. Tissue reactions in air were somewhat reduced but in OHP these were very severe and beyond therapeutic tolerance and of the same order as for a single dose of 3000 rads (OHP). At this dose level in OHP, 3 × 1000 rads in 8 days, now produced a gain in therapeutic ratio, namely, no reduction in cure rate (75 per cent) but a lessening of the tissue reaction index from 5·8 to 2·8, which represents a marked qualitative change from necrosis to epilation and moderate atrophy, and clearly superior to the therapeutic ratio for 2 × 1500 rad fractions in OHP. Attention is drawn to 3 × 1000 rads in OHP over 8 days (cure rate 75 per cent, tissue reaction index 2·8) and 3 × 1000 rads in air over the same 8 days (cure rate zero per cent, tissue reaction index 2·0).

(*d*) 6000 *rad group with tourniquet anoxia*. (Fig. 8).—The observations in this group are limited but suggest that fractionation of this large dose under anoxic conditions to produce a substantial cure rate (average 85 per cent), reduced the tissue reaction level from approximately 4·5 (single dose 6000 rads with anoxia) to approximately 2·1 and again demonstrates a similar gain in therapeutic efficiency, with a halving of tissue reactions. This is virtually identical to the situation for fractionation in OHP, at the 2000 rad dose level, and clearly supports the results for oxygen effect obtained with single doses, i.e. a DRF of 3·2. In this group further fractionation (3 increments) again produced zero cure rate.

Fig. 7.—3000 rad fractionated treatments. (OHP = irradiation in 30 psi gauge pressure oxygen, Ano. = tourniquet compression proximal to tumour during irradiation in air).

Fig. 8.—6000 rad fractionated treatments in air with tourniquet constriction to produce tumour anoxia.

*Homograft effects*

In a previous report (van den Brenk, 1961*a*) it was shown that for this tumour-host system, whole body irradiation of host mice with 376 rads twenty-four hr. preceding inoculation reduced the $ED_{50/50}$ cell dose from 281 to <10 cells. If 7 days elapsed between irradiation and inoculation, apparent partial immunological recovery resulted in an $ED_{50/50}$ cell dose of 31 cells. In a limited and preliminary experiment, one week old tumours, representing a calculated tumour cell population of approximately $N = 0{\cdot}32 \times 10^8$ cells (van den Brenk, 1961*a*) were left either untreated, or irradiated *in situ* (1000–3000 rads single dose in OHP). The following day (i.e. 8 days after inoculation, 1 day after local treatment), $10^2$–$10^5$ freshly harvested EAT cells were inoculated in the contralateral (left) hind limb of the mice and the tumour incidence scored for both limbs. The results (Table III) show that the $ED_{50/50}$ was of the order of $10^3$–$10^4$ cells, and

TABLE III.—*Tumour Incidence in Right and Left Limbs of Mice, in which the Right Limb was Inoculated with* $10^6$ *EAT Cells, 24 Hr. after* 400 *Rads Whole Body Irradiation was given to the Host, and the Growing Tumour Treated by Irradiation in OHP or with OHP Only* 7 *days After the Inoculation. One Day After the Treatment of Right Legs, the Left Limb was Inoculated with Freshly Harvested (Unirradiated) EAT Cells in Doses of* $10^2$–$10^5$ *Cells. Palpable Tumours Scored in Both Limbs* 9 *Weeks After First Inoculation (Right Limb) of the Mice*

| Group (treatment to right leg and number of mice used) | Challenge inoculum left leg (number EAT cells) | Tumour incidence at 8 weeks (percentage) | | Surviving fraction at 8 weeks |
|---|---|---|---|---|
| | | Right leg (treated) | Left leg* (untreated) | |
| I. Mock irradiation in 30 psi $O_2$ (12) | $10^2$ cells | 6/6 (100) | 2/6 (33) | 6/12 |
| II. Mock irradiation in 30 psi $O_2$ (12) | $10^3$ ,, | 7/7 (100) | 2/7 (29) | 7/8 |
| III. Mock irradiation in 30 psi $O_2$ (6) | $10^4$ ,, | 5/5 (100) | 2/4 (50) | 5/6 |
| IV. Mock irradiation in 30 psi $O_2$ (8) | $10^5$ ,, | 5/5 (100) | 5/5 (100) | 5/6 |
| V. 1000 rads in 30 psi $O_2$ (12) | $10^2$ ,, | 7/11 (64) | 1/11 (9) | 11/12 |
| VI. 3000 rads in 30 psi $O_2$ (12) | $10^2$ ,, | 0/7 (0) | 2/7 (29) | 7/12 |
| VII. 2000 rads in 30 psi $O_2$ (8) | $10^3$ ,, | 0/3 (0) | 0/3 (0) | 3/8 |
| VIII. 2000 rads in 30 psi $O^2$. (8) | $10^4$ ,, | 2/5 (40) | 3/5 (60) | 5/8 |
| IX. 2000 rads in 30 psi $O_2$ (8) | $10^5$ ,, | 0/5 (0) | 5/5 (100) | 5/8 |

* All tumours showed active, progressive growth, and none regressed.

treatment of the right limb made little difference. Whilst the participation of homograft reactions seems clear, several factors make interpretation difficult in this complex *in vivo* experiment. It has been shown that for dual tumours in the one host, homograft reactions and immunological attenuation lead to complex results (van den Brenk, 1961*c*), and in the unirradiated host the $ED_{50/50}$ cell dose has to be increased to over 3000 cells for the production of two tumours per mouse. In addition other abscopal effects attributable to whole body irradiation (nutritional status, infection etc.) may contribute to cell death, and the " Révész " effect (Révész, 1958) may also be a factor in promoting cell survival in irradiated solid tumours which is not obtained for the test inoculations designed to expose immunological factors. Further experiments along these lines seem indicated not only for homograft situations but also for isologous and even autologous tumour-host relationships.

### DISCUSSION

The experiments of Hewitt (1958), Deschner and Gray (1959) and Hornsey and Silini (1961), employing relatively exact quantitative techniques, have established the importance of oxygen effect in tumour radiosensitivity. Other animal studies (Hollcroft, Lorenz and Mathews, 1952 ; Dittrich and Stuhlman, 1954 ; du Sault, Eyler and Dobben, 1959 ; Thomlinson 1960, 1961 ; and van den Brenk, 1961*a*) have shown similar dependence on oxygen, of intact solid tumours irradiated *in vivo*. The earlier studies of Gray *et al.* (1953) focussed attention on the therapeutic importance of oxygen effect in clinical practice and also inspired the important clinical project of Churchill-Davidson, Sanger and Thomlinson (1957) in which an unconventional fractionation policy was adopted and dictated by the complexities of the technique employed, using high pressure oxygen. Since fractionation of dose is almost universal in clinical radio-therapeutic practice, the results are difficult to interpret from the statistics available, and an experimental approach seems desirable, and even more so in the light of this more recent interest in oxygen effect. The argument has been raised from time to time, that a gradual improvement in the haemodynamics of solid tumours during a fractionated course of irradiation results, and that tumour oxygen tensions rise *parri passu* and largely preclude the necessity of artificially equating tumour and tissue oxygen tensions. This view was supported by the experiments of Cater (1958) in humans, in which direct measurement of oxygen tensions in tumours and normal tissues were made polarographically before, during and after radiotherapy. On the other hand, du Sault *et al.* (1959), using fractionated treatments in spontaneous $C_3H$ mouse mammary adenocarcinoma reported that fractionation decreased radio-curability of such tumours, whilst breathing 95 per cent $0_2$/5 per cent $CO_2$ enhanced radio-curability. Since these studies were limited, and failed to make an adequate quantitative assessment of tissue reactions and therapeutic ratio, it was decided to reinvestigate this problem in the immunologically attenuated mouse using growths of solid Ehrlich tumours, not only treated with single and fractionated doses in air, but also in high pressure oxygen (OHP) and with tourniquet induced anoxia, along the lines of a previous experiment (van den Brenk, 1961*a*).

In the experiments, reported above, the rapidity of growth of the untreated and recurrent tumours, precluded extension of the overall fractionation time beyond 8 days, and repeated anaesthesia necessary made 3 fractionated treatments the useful maximum. A repeat of single dose experiments confirmed the oxygen effect previously obtained, and for tumour cure rate the DRF of 3·2 for irradiation with 200 kv X-rays of the solid tumour *in vivo*, parallels closely that of 3·1 obtained by Hornsey and Silini (1961) for the near tetrapoloid EAT *in vitro* inoculated in new born mice and using 200 kv X-rays. However, in these present experiments, cure rates in air were higher ($LD_{50}$ dose decreased from 4000 r to approximately 2500 rads), and is attributed to reduced depth of anaesthesia and experience in the technique lessening interference with limb oxygen tensions. A more accurate quantitative assessment of normal tissue reactions has confirmed the marked effect of OHP in increasing normal tissue radiosensitivity in mice, and similarly, tourniquet anoxia was shown to cause marked reductions in tumour cure rate ($LD_{50}$ = 4620 rads) and skin reactions. A calculation of the respective DRF's for tumour and normal tissue (3·2 and 2·7, respectively, for OHP treatments relative to anoxia induced by tourniquet compression) led to the conclusion that there was little alteration in therapeutic ratio, but it was suspected that diffusion of oxygen through the skin, shown by Chase and Hunt (1959) to contribute substantially to radio-sensitivity of mouse skin, complicated the interpretation. This was substantiated by showing that tourniquet compression in mice in OHP or air, both resulted in reductions in tumour cure rates to the anoxic level, but that in the OHP mice the tissue reactions were substantially increased. Within the limits of experimental accuracy it could be deduced that 30 psi oxygen contributes to radiosensitivity of skin by direct surface diffusion through skin, an oxygen tension approximately equal to the amount reaching skin, in mice breathing air at ambient pressure, by diffusion from both blood and the surface, and that in the latter animals, radiosensitivity of the integument is at approximately half the fully oxygenated level. It follows that it is quite incorrect to translate therapeutic ratios obtained in the mouse, under the various conditions of OHP, air breathing and tourniquet anoxia, to large animals and man, where oxygen diffusion through the integument is probably less and where the gain in therapeutic ratio would be substantially higher. Attention has already been directed to another factor which decreases oxygenation of the integument in air, namely, anoxia caused by prolongation of anaesthetic times for higher doses, which resulted in a decrease in slope of the " air " tissue reaction/dose relationship (Fig. 2). This effect would also tend to give a higher therapeutic ratio in air treated tumours, than either that for OHP or tourniquet anoxia.

The fractionation results have clearly shown that oxygen effect is equally important in this system for fractionated and for single treatments. A summary of some relevant tumour cure rates and tissue reactions in support is shown (Table IV), and the fractionated doses have been corrected for a 2-hit survival curve ($D_{37}$ = 130 rads in oxygen, DRF = 3) in Fig. 9, 10 and 11.

TABLE IV.—*Effect of Oxygen on Tumour Cure Rates and Tissue Reactions for Various Fractionated and Single Treatments*

| Treatment | Tumour cure rate (per cent) | Tissue reaction index |
|---|---|---|
| 1 × 2000 rads (OHP) | 67 | 4·0 |
| (8 days) 2 × 1000 „ „ | 57 | 1·0 |
| (8 days) 3 × 1000 „ „ | 75 | 2·9 |
| 1 × 2000 „ (Air) | 27 | 2·1 |
| 1 × 3000 „ „ | 50 | 3·3 |
| (8 days) 2 × 1000 „ „ | 0 | 0·9 |
| (8 days) 3 × 1000 „ „ | 0 | 2·0 |
| 1 × 6000 „ (Anoxia) | 70 | 4·4 |
| (8 days) 2 × 3000 „ „ | 78 | 2·0 |
| (8 days) 3 × 2000 „ „ | 0 | 1·7 |

It is seen that 2 × 3000 rads in 8 days (anoxia) is approximately equivalent in respect to tumour cure rate as 3 × 1000 rads (OHP) and tissue reactions are also similar (Fig. 9)—a result consistent with a DRF of 3·2 obtained for single doses. Similarly 3 × 2000 rads in 8 days (anoxia) giving zero cure rate and tissue reaction 1·7, is comparable to 3 × 667 rads in 8 days (OHP) giving 12 per cent cures and a tissue reaction 1·9 (Fig. 6 and 9). It is difficult to reconcile these

FIG. 9.—Results for tumour cure rates and tissue reactions plotted for fractionated treatments in OHP and tourniquet anoxia after correcting the doses for a 2-hit survival curve. The curves drawn are the regressions for single dose results. Numbers against points are the overall fractionation time in days.

results, and particularly those for equal and unequal dose fractions in OHP and air, with progressive oxygenation resulting from tumour destruction and improved haemodynamics during treatment. On the other hand the various results show a clear relationship of tumour cell population and dose effect to oxygen dependence, and that rapid proliferative integrity appears within a short time after inadequate irradiation. This is in accordance with the *in vitro* results of Elkind and Sutton (1959) who showed for Chinese hamster tissue that a dose of 505 rads is followed by a division delay of about 30 hr. after which surviving cells are fully recovered in respect to proliferation and clone formation. These authors suggest that failure to recognise this rapid recovery in fractionation techniques may lead to an erroneous (low) estimate of cellular populations. However, the relative increase in survivors following a fractionated treatment, based on dose-survival curves, must be taken into account. For example, a tumour comprising $10^8$ cells is reduced to approximately 20 cells by a single dose of 2000 rads in oxygen (based on Hornsey and Silini's data for EAT), whilst $2 \times 1000$ rads under similar conditions and assuming no cellular addition due to proliferation during the fractionation period would result in approximately 400 cells. Assuming an $ED_{50}$ of 100–200 EAT cells in the immunologically attenuated mouse, the difference in cure rate for the two treatments would not be surprising. Similarly for $3 \times 667$ rads the calculated survival would be more than $10^5$ cells for irradiations in oxygen and any cures *in vivo* would be due to indirect cellular or abscopal effects for this

FIG.10.—Fractionation results for air treatments, corrected as in Fig. 9 using the $D_{37}$ $(1/\lambda)$ = 130 rads for oxygen. Curves are single dose regressions.

system. However, apart from this aspect, cellular repopulation discussed above must be taken into consideration, and clinical observations in animals made during a fractionated course of irradiation showed measurable and progressive increase in size of tumours, particularly with fractions of less than 1000 rads in OHP and air, and with 2000 rad fractions under anoxic conditions. That similar cellular recovery in normal tissues contributed to the improvement in therapeutic ratio observed for certain fractionated treatments, particularly for OHP treatments, seems probable although whether such recovery is due to cellular repopulation or other physiological aspects of repair and tissue recovery remains to be elucidated.

The apparent lack of tumour oxygenation during progressive cellular depletion with fractionation reported above, requires further consideration in respect to physiological characteristics of the host animals, tumour vascularisation and radiation changes. The mouse tissues are at a disadvantage in respect of oxygenation ; the haemoglobin saturation curve is relatively flat and displaced to the right (Schmidt-Neilson and Larimer, 1958) such that blood is only 70–75 per cent saturated with oxygen at 100 mm. Hg. Moderate reductions in respiratory exchanges would result in relatively lower tissue oxygen tensions than in man and larger animals, a result further potentiated by the high tissue oxygen consumptions in the mouse. It is not surprising that moderate reductions in respired oxygen tension (7–10 per cent) in the mouse results in substantial whole

FIG. 11.—Fractionation results for divided doses administered in air and OHP alternatively and corrected as in Fig. 9 and 10.

body protection against radiation damage (van der Meer and van Bekkum, 1959). Furthermore, the relative lack of myohaemoglobin in this animal fails to provide a " store " of oxygen to feed back into the blood during conditions of induced general or local anoxia. If further consideration is given to the marked effect of slowing of capillary blood flow on cellular oxygenation, even in respect to OHP exposure, and to the unstable, poorly developed and bizarre form of vascular bed seen in the solid Ehrlich tumour, it is not surprising that tissue anoxia and particularly tumour anoxia is readily induced by such simple expedients as anaesthesia, slight local pressure or stretching, hypoventilation, mild stresses and other experimental procedures. It has been reported that tissue pressures in tumours are abnormally high (Young, Lumsden and Stalker, 1950), and if this contributes to circulatory stasis as growth progresses, improved oxygenation could be expected from cell loss after irradiation. However, there is also often reason to suspect that growth of blood vessels (angiogenesis) in tumour growth is defective and limited and contributes to tumour necrosis. The initiating factor for angiogenesis in a proliferating tissue is probably general metabolic activity and nutritional requirements—a view so clearly expounded last century by Virchow in his studies on cellular pathology (Virchow, translated version, 1958). Acute and chronic observations of mature and proliferating blood vessels following irradiation *in vivo*, (van den Brenk, 1956, 1957, 1959) have failed to show convincing evidence of capillary circulatory changes conducive to better oxygenation of adjacent tissues. Also, metabolites such as histamine and 5-hydroxytryptamine, exogenously administered and endogenously liberated, cause radioprotection by anoxic mechanisms (van den Brenk and Elliott, 1958 ; van der Meer and van Bekkum, 1959). Again, the relationship of tissue tension to haemodynamics is complex (Wright, 1937 ; Nichol *et al.*, 1951 ; van den Brenk *et al.*, 1956), and the hypothesis that blood flow is passively dependent on tissue tension is a gross over-simplification. A common clinical experience in humans is that during a course of radiotherapy, large, solid, pulsating tumours show a reduction in pulsation and heat as regression occurs—a finding for which Virchow's hypothesis provides a more plausible explanation than simple mechanical theories. Nevertheless, this whole problem is of sufficient importance to warrant extensive investigations—both experimental and clinical.

Whilst homograft effects cannot be excluded from the present investigation, it is considered unlikely that these significantly affect the conclusions drawn in respect to oxygen effect and fractionation for these experiments. Spontaneous tumours would appear a more desirable experimental medium, but cell populations are even more difficult to determine and there exists considerable histopathological variability in individual tumours, as well as differences in growth rates, vascular pattern and degrees of differentiation. Examples of the latter such as cornification, acinar and alveolar patterns and fibrillogenesis, makes estimation of the proliferative cell population difficult and separation of cells for titration experiments is often impossible. Furthermore, the topography of such tumours often presents technical difficulties and in our experience, for the control of tumour dosimetry and oxygen tension, hind limb transplants provide the most satisfactory solution. Whilst benzopyrene induced tumours in hind limbs of mice are at present being investigated as an experimental trial, such tumours are also suspect on immunological grounds (Prehn and Main, 1957) and show unpredictable variations in respect to histological structure and rate of growth, and their site of origin cannot be accurately designed in small animals.

### SUMMARY

Solid hyperdiploid Ehrlich ascites tumours growing in the hind limbs of hybrid mice, immunologically attenuated by previous whole body irradiation, were locally irradiated by single and fractionated doses of 250 kv X-rays. During the various irradiations, tumour and tissue oxygen tensions were varied by (I) breathing high pressure oxygen (OHP) at 30–45 psi gauge pressures (II) breathing air at ambient pressure and (III) with blood flow to the affected limb arrested by tourniquet compression.

Oxygen enhancement factors (dosage reduction factors) of 3·2 and 1·5 were obtained for OHP and air respectively, for tumour cure rate assessed 8 weeks after irradiation. Oxygenation also increased normal tissue damage due to irradiation by factors of 2·7 and 1·7 for OHP and air respectively.

Fractionation of the dose decreased both cure rate and tissue reactions for all combinations investigated, for periods up to 8 days. However, for certain fractionation treatments the reduction in tissue reactions was relatively greater and gave an enhancement in therapeutic ratio and effectiveness.

It was shown that in mice, the diffusion of oxygen through the integument from the surface, is highly significant and tends to give an erroneous conception of the value to be derived in respect to therapeutic ratio, from equating tumour and tissue oxygen tensions by artificially raising or lowering oxygen levels in the animal.

Fractionation experiments failed to demonstrate that initial irradiations induce better tumour oxygenation which precludes the necessity for artificially equating tumour and tissue oxygen tensions at subsequent fractional treatments.

### REFERENCES

van den Brenk, H. A. S.—(1956) 'Progress in Radiobiology'. Edinburgh (Oliver and Boyd), p. 386.—(1957) *Proc. Coll. Radiol. Aust.*, **1**, 29.—(1959) *Amer. J. Roentgenol.*, **81**, 859.—(1961*a*) *Brit. J. Cancer*, **15**, 61.—(1961*b*) *J. Coll. Radiol. Aust.*, **5**, 113.—(1961*c*) *Brit. J. Cancer*, **15**, 798.

*Idem*, Cass, N. M. and Chambers, R. D.—(1956) *Brit. J. Anaesth.*, **28**, 98.

*Idem* and Elliott, K.—(1958) *Nature, Lond.*, **182**, 1506.

Cater, D. B.—(1958) *Rep. Brit. Emp. Cancer Campgn.*, **35**, 479.

Chase, H. B. and Hunt, J. W.—(1959) 'Pigment Cell Biology'. New York (Academic Press Inc.), p. 537.

Churchill-Davidson, I., Sanger, C. and Thomlinson, R. H.—(1957) *Brit. J. Radiol.*, **30**, 406.

Deschner, E. E. and Gray, L. H.—(1959) *Radiation Res.*, **11**, 115.

Dittrich, W. and Stuhlman, H.—(1954) *Naturwissenschaften*, 41, 122.

Elkind, M. M. and Sutton, H.—(1959) *Nature, Lond.*, **184**, 1293.

Finney, D. J.—(1951) 'Probit Analysis'. 2nd Edition. London (Cambridge University Press).

Goldacre, R. J. and Sylvén, B.—(1959) *Nature, Lond.*, **185**, 63.

Gray, L. H., Conger, A. D., Ebert, M., Hornsey, S. and Scott, O. C. A.—(1953) *Brit. J. Radiol.*, **30**, 406.

Hewitt, H. B.—(1958) *Brit. J. Cancer*, **12**, 378.

Hollcroft, J., Lorenz, E. and Mathews, M.—(1952) *J. nat. Cancer Inst.*, **12**, 751.

Hornsey, S. and Silini, G.—(1961) *Int. J. Radiation Biol.*, **4**, 135.

van der Meer, C. and van Bekkum, D. W.—(1959) *Ibid.*, **1**, 5.

Moore, R.—(1961) *Radiation Res.*, **14**, 296.

Nichol, J. T., Girling, F., Jerrard, W., Claxton, E. B. and Burton, A. C.—(1951) *Amer. J. Physiol.*, **164**, 330.

Prehn, R. T. and Main, J. M.—(1957) *J. nat. Cancer Inst.*, **18**, 769.

Révész, L.—(1958) *Ibid.*, **20**, 1157.

du Sault, L. A., Eyler, W. R. and Dobben, G. D.—(1959) *Amer. J. Roentgenol.*, **82**, 688.

Schmidt-Neilsen, K. and Larimer, J. L.—(1958) *Amer. J. Physiol.*, **195**, 424.

Thomlinson, R. H.—(1960) *Brit. J. Cancer*, **14**, 555.—(1961) 'Brookhaven Symposium in Biology', **14**, 204.

Virchow, R.—(1958) 'Cellular Pathology' translated by F. Chance. London (New Sydenham Society), p. 72, 84, 123, 246

Wright, R. D.—(1937) *Aust. N.Z. J. Surg.*, **7**, 215.

Young, J. S., Lumsden, C. E. and Stalker, A. L.—(1950) *J. Path. Bact.*, **62**, 313.